# Comments on the mechanisms of action of radiation protective agents: basis components and their polyvalence

**DOI:** 10.1186/2193-1801-3-414

**Published:** 2014-08-07

**Authors:** Mikhail V Vasin

**Affiliations:** Department of Medicine of Catastrophe, Russian Medical Academy of Post-Graduate Education, St. Polikarpova 10, 125284 Moscow, Russia

**Keywords:** Radioprotector, Radiomitigator, Radiomodulator, Mechanism of action

## Abstract

**Purpose:**

These comments suggest a division of radiation protective agents on the grounds of their mechanism of action that increase the radio resistance of an organism.

**Conclusion:**

Given below is the division of radiation protective agents on the basis of their mechanism of action into 3 groups: 1) Radiation protective agents, with the implementation of radiation protective action taking place at the cellular level in the course of rapidly proceeding radiation-chemical reactions. At the same time, when the ionizing radiation energy is absorbed, these agents partially neutralize the “oxygen effect” as a radiobiological phenomenon, especially in the radiolysis of DNA; 2) Radiation protective agents that exert their effect at the system level by accelerating the post-radiation recovery of radiosensitive tissues through activation of a number of pro-inflammatory signaling pathways and an increase in the secretion of hematopoietic growth factors, including their use as mitigators in the early period after irradiation prior to the clinical development of acute radiation syndrome (ARS). 3) Radiomodulators including drugs and nutritional supplements that can elevate the resistance of the organism to adverse environmental factors, including exposure to ionization by means of modulating the gene expression through a hormetic effect of small doses of stressors and a “substrate” maintenance of adaptive changes, resulting in an increased antioxidant protection of the organism. Radiation protective agents having polyvalence in implementation of their action may simultaneously induce radioprotective effect by various routes with a prevalence of basis mechanisms of the action.

## Introduction

Clinical radiotherapy of tumors demands a knowledge of the potential effectiveness of radiation protective agents to reduce radiation damage to healthy tissues, as well as knowledge of their impact on the radiosensitivity of tumor tissues. A theoretical understanding of the mechanisms of the radiation protective action of the agents may contribute to this knowledge.

The key aspects of radiation protective agents are the practical need to use them in specific scenarios of radiation exposure and the corresponding tactical and technical requirements for medical preparations. These aspects are related to the indications for use (depending on the nature and severity of a radiation injury), possible routes of administration, acceptable side effects in certain situations, pharmacodynamic characteristic, as well as the possibility of expected scheme of repeated administration.

A recent monograph (Arora et al. 2008) and a review (Weiss and Landauer 2009) provide a contemporary view of the pharmacological measures against the harmful effects of ionizing radiation, as well as comprehensive information about radiation protective agents — their classification, the history of discovery, pharmacological and radiation protective properties, data on tolerability, and their current use. Based on the current knowledge of the mechanism of action of radiation protective agents, in particular gene regulation induced by the increased radiation resistance of the organism, this commentary mainly focuses on some of the common features typical for their mechanism of action. For most of these agents applied in practical medicine it is possible to distinguish three main pathways for the implementation of the mechanism of action, each of which has their own specificity in predetermining the pharmacodynamic properties and possible methods in their practical application (Vasin 1999, 2012).

## Review

***Radioprotectors (chemical protection by Zenon Bacq) that exert their effect on physicochemical and biochemical levels in cells during exposure to ionizing radiation through partial neutralization of radiosensitizing oxygen effect***

*The mechanism of protective action of the radioprotectors from the family of aminothiols*

Radioprotectors realize radiation protective action at the cellular level in the course of rapidly proceeding radiation-chemical reactions. At the same time, when the ionizing radiation energy is absorbed, these agents partially neutralize the “oxygen effect” as a radiobiological phenomenon, especially in the radiolysis of DNA (Hutchinson 1961). The essence of the “oxygen effect” which finds its manifestation at different levels of the biological organization in the world of plants and animals lies in the increased radiation damage of DNA, membranes, proteins, carbohydrates in the presence of oxygen and transition valence ions (Fe^+2^, Cu^+^, Zn^+2^). Reactive oxygen species are formed as a result of radiation-chemical reactions: superoxide anion radical (О_2_^**¯**^•), hydroperoxide radical (НО_2_•), atomic oxygen, oxygen in the long-lived excited form, singlet oxygen (О_2_**´**).

According to the oxygen fixation hypothesis, radicals in DNA induced by indirect action via aqueous radicals may be fixed by reaction with oxygen or chemically repaired by H-atom donation from antioxidants such as sulphydryl compounds (Hall and Giaccia 2012).

The mechanism of the action of radioprotectors is achieved either by a pharmacological reduction of the oxygen content in the cell or by a direct participation of thiol groups of sulfur radioprotectors in competitive reactions with oxygen for the DNA radical.

**Figure 1:**
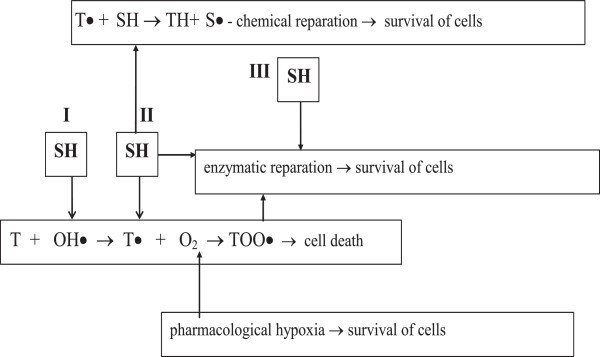
**Scheme for realization and neutralization of the oxygen effect at the level of cell.** Note: T – targets.

The main role in the realization of radioprotective properties of the known most active sulfur-containing radioprotectors at the level of radiation-chemical reactions is assigned to their ability to interact directly with the radiolysis products of macromolecules, thus causing their reparation (reaction of type II in Figure 1), rather than to the competitive relationships with macromolecules for the radiolysis products of water.

These radioprotectors exerting their effects by this mechanism include sulphur-containing radiation protective drugs that are β-mercaptoethylamines, aminoalkyl thiosulphates, aminoalkyl dithiophosphates, aminoalkyl isothiouronium, and thiazolidine and thiazoline derivatives.

The protective effect of sulfur-containing radioprotectors is associated with a free SH-group in the composition of their molecule. The possibility to transfer the hydrogen atom from the SH-group of the protector to the radical of the macromolecules provides its chemical reparation: М• + HS–R → MH + R–S• (Alexander and Charlesby 1954).

Chemical reparation of the radical of deoxyribose in DNA by a hydroxyl radical under dehydrogenation of the molecule is of crucial importance for the reduction of the frequency of chromosomal aberrations in the cells under the influence of radioprotectors:

deoxyribose (-H)• + RSH = deoxyribose + RS• (Fahey 1988; Held 1988).

The rate constants for the transfer of a hydrogen atom from cysteamine to the radical of a macromolecule are close to 10^6^ – 4•10^8^ М^-1^•с^-1^ (Ormerod and Riesz 1967). The known sulfur-containing radioprotectors in the reactions of interaction with the radicals of macromolecules and DNA reach the radiation protective effect at the concentrations ~ 2 - 3 times lower than it is possible under the influence of endogenous thiols.

This phenomenon is mainly associated with the positive charge of aminothiols due to the presence in a molecule of the amino group, which should be located no further than 2 - 3 carbon atoms from the free thiol group of the radioprotector to provide the optimum effect of the particular chemical structure. Due to the positive charge of their molecule and by means of electrostatic attraction aminothiols can closely interact with the DNA molecule bearing a negative charge (Zheng et al. 1988, 1992; Spotheim-Maurizot et al. 1991).

However, the interaction of aminothiols with the DNA molecule can have a more specific character than a simple adsorption on a macromolecule. Jellum (1965) was the first to show the possibility of forming a chemical complex between cysteamine and DNA through a diamine link. Due to a high positive charge of the disulfide WR-1065 (Z = +4) on the surface of DNA the amount of the radioprotector fixed through a diamine bond is 3 times higher than by means of its electrostatic adsorption (Newton et al. 1996).

Neutralization of the negative charge of DNA during the formation of a new complex of disulfide WR-1065 with DNA leads to the contraction of the DNA molecule along the axis with the formation of a liquid crystal spring-like structure with the narrowing of the channels between the DNA helices (Savoye et al. 1997). The latter mechanism can be effective for the reduction of the radiation damage to DNA due to the decrease in the number of the sensitive places to be attacked by the hydroxyl radical at a density of fixation of more than one molecule of the protector per 4 nucleotides of DNA (Savoye et al. 1997).

Since the radioprotector action is competitive with the ionizing radiation effect, its radioprotective efficacy may be detected by a dose reduction factor (DRF), which is defined as the ratio of radiation 50% lethal dose (LD50) for the drug-treated, irradiated animals to the LD50 for irradiated control animals. This exponent is the most informative for comparative quantitative evaluation of potential of radiation protective agents.

**Figure 2:**
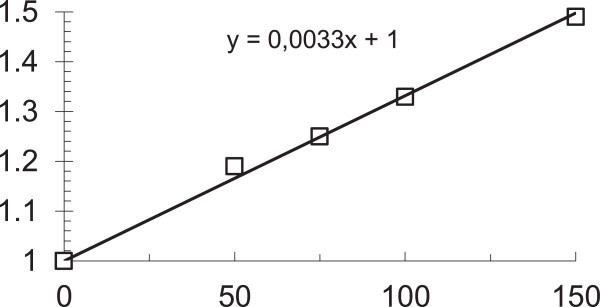
**Dose-response relationship of radiation protective effect of cystamine in the experiment on mice (Vasin et al.** 1970 **).** Abscissa: the dose of cystamine, mg/kg. Ordinate: radiation protective effectiveness of cystamine, DRF.

The realization of radioprotective action of sulphur containing radioprotectors through physicochemical reaction with products of radiolysis predetermines a direct correlation between the level of expression of radiation protective efficacy of aminothiols in terms of DRF and the administered dose of the preparation and its concentration in the radiation-sensitive tissues (Patt et al. 1953; Doherty 1960; Koch 1967; Hasegawa and Landahl 1970; Vasin et al. 1970) (Figure 2).

The efficacy of radioprotectors is limited threshold that cannot exceed the radio-sensitising action of oxygen dissolved in tissues, which increases radiation damage of the cells by 2- to 3-fold in the term of DMF. Similar high protective effect with DRF being equal 3 was observed at radioprotective composition of cysteamine, AET, glutathione and serotonin in the experiment on mice (Maisin et al. 1968).

The highest activity of radioprotectors takes place in the hematopoietic tissue and salivary glands (DRF = 1.5 – 2.0), to a lesser extent in the skin (DRF up to 1.5), intestines (DRF up to 1.3) and testis (DRF = 1.1 – 1.2) (Travis 1984).

The radioprotective effects of aminothiols is first of all associated with the reduced radiation damage to stem cells of bone marrow, intestine and skin (Duplan and Fuhrer 1966; Smith et al. 1966; Vasin et al. 1977). Comparative studies in vitro showed protecive effect amifostine in the term of DRF for the cells forming colonies of myeloid, megakaryocyte and erythroid lineages, as well as bone marrow fibroblasts was close to 2 (Ramdas et al. 2003).

**Figure 3:**
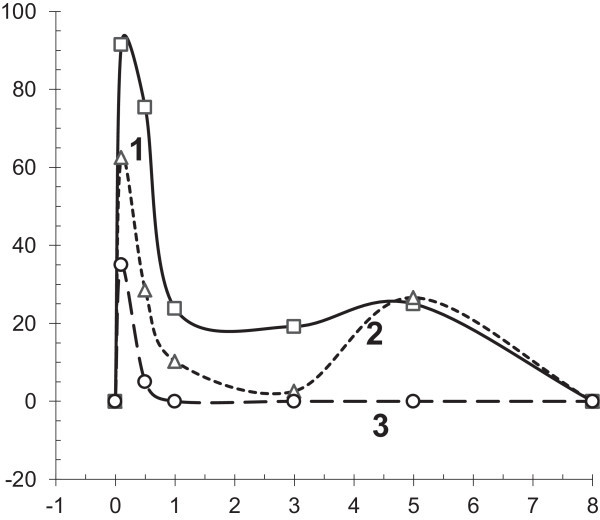
**Radioprotective effect of cystamine administrated IP at various periods before irradiation in the experiment on mice (Vasin et al.** 1970 **).** Abscissa: the periods of cystamine administration before irradiation, hours. Ordinate: radioprotective effect of cystamine at LD_95/30_ of gamma-irradiation, %. 1–150 mg/kg, 2–100 mg/kg, 3–75 mg/kg.

Radioprotectors are of great practical interest because of their ability to rapidly (within minutes) increase the resistance of the cells to radiation exposure as well as their ability to protect against high levels of radiation, including exposures at super-lethal doses (up to 10-15 Gy), both of which are absent in other types of radiation protective agents. The time needed to implement the radiation protective action of this group of compounds is within 1 to 2 minutes after administration beginning from a moment of an incorporation of aminothiol molecules in cells. The short duration of maximum action of the radioprotectors is associated with a sufficiently high rate of metabolism in the body and is generally limited to one hour (Vasin et al. 1970) (Figure 3). The time of an elimination of aminothiols from a radiosensitive tissues and decrease of their optimal radioprotective action is identical (Vasin et al. 1971).

However, small protective effect of aminothiols prolongs over several hours after their administration that can be induced by biochemical effects of aminothiols. As shown in Figure 3, the appearance of protective effect of aminothiols in six hours after their administration is possibly induced through an activation of Mn-SOD by the radioprotector (Murley et al. 2006, 2007). Pharmacological properties of aminothiols can determinate complex of poly-functional actions that is significant for the radiation protection, including the influence to mitochondria respiration following acute hypoxia, gene Mn-SOD expression, activity of the Tip60 acetylransferase, etc.

The decrease of DNA damage by aminothiols is basic factor in their protective action. Aminothiols can inhibit DNA synthesis and cell division (Vaughan et al. 1989; Chigareva et al. 1990). Cystamine molecules can block an activity of some thiol-dependent enzymes: caspase-3 (Lesort et al. 2003), transglutaminase (Jeitner et al. 2005) that tie together enzyme molecules. It is likely that the conformational structure of DNA undergoes some changes under the influence of aminothiols associated with the condensation and stabilization of chromatin, thus enhancing the interplay between the transcriptional factors (nuclear factor - kappaB, protein-1 activator and tumor supressor p53) and DNA, which leads to the subsequent activation of a number of genes. One of the constituents of this reaction is the activation of the p53 tumor suppressor under the action of aminothiols. This reaction develops given the interaction of extracellular thiols with the cell membrane. The transfer of the stress signal to p53 is carried out through the c-Jun N-terminal kinase (Pluquet et al. 2003). In addition, aminothiols due to their ability to block topoisomerase II, as well as under activation of p53 through the inhibitor of cycline-dependent kinases p21waf-1 prevent the passage of cells through the cell cycle in the G1 phase. Mitotic block over six-eight hours provide more opportunities for the reparation of the DNA from “hidden” potential radiation damage (Murley and Grdina 1995; North et al. 2000). The activation of nuclear factor – kappaB by amifostine also inhibits apoptosis of hematopoietic progenitor cells (Romano et al. 1999). These processes can be cause of radiation mitigative effects of aminothiols at their administration after irradiation that more extensively develop in the condition of non-uniform irradiation or partial shielding of the body (Maisin et al. 1953a, [b]; Shashkov et al. 1971; Vasin 2012). That is possibly connected with the stimulatory effect of aminothiols on the hematopoietic stroma elements (Romashko et al. 1990).

Radioprotective properties of aminothiols is associated with their ability to raise the level of reduced equivalents in the cells and, in the first, endogenous thiols: glutathione and cysteine - from 2-3 up to 10 times (Wardman et al. 1992). The elevated level of endogenous thiols under the influence of aminothiols causes the development in the cell of the “redox stress” or “biochemical shock” (according to the terminology of Z. Bacq), which, in particular, manifests itself as the swelling of mitochondria in the cells (Lehninger and Schneider 1959; Neubert and Lehninger 1962). The swelling of mitochondria is directly regulated by the state of the thiol-disulfide equilibrium in the respiration chain. Under conditions of the mitochondrial swelling, the number of SH-groups increases.

The changes in the structure of mitochondria in the form of swelling are directly reflected in the operation of the phosphorylation respiratory chain, namely, in the degree of relationship between the processes of electron transport and oxidative phosphorylation, which is measured by the P/O ratio. The maximum values of the P/O ratio are observed when the integrity of mitochondria is the highest. When mitochondria swell, the distance between the outer and inner membrane decreases and the flow of electrons through the membranes accompanied by the release of energy in the form of heat due to the intensification of the processes of free oxidation increases.

The reduced level of the P/O relationship represents itself one of the ways of regulating the respiratory chain. A mild and moderate uncoupling of oxidative phosphorylation is a necessary condition for the acceleration of oxidation when there is a physiological requirement for increased energy. Extensive uncoupling of oxidative phosphorylation with sharp oxygen consumption in cells induces to the development of acute cellular hypoxia that is key factor for an implementation of high radioprotective effect. This fact was confirmed by the administration of uncoupler of oxidative phosphorylation 2,4-dinitriphenol when it was detected 100% protective effect (Praslicka et al. 1962; Vacek and Rotkovska 1964).

Aminothiols have an effect on respiration and phosphorylation in cells, which is accompanied by mitochondrial swelling and intensification of free oxidation processes (Lelievre 1965; Firket and Lelievre 1966; Skrede 1966; Vladimirov and Libikova 1970). Amifostine added to the medium of mammalian cell culture rises rapidly oxygen consumption and the temperature of the tissue culture in vitro (Purdie et al. 1983). Cystamine also increases cellular respiration of hepatocytes (Iarmonenko 1961) and erythrocytes in vitro (Kuznetsov and Tank 1964). The administration of cystamine in humans at a dose of 0.6 g was found to increase the oxygen consumption by 9% and body temperature by 0.2 – 0.3°C for 1 – 2 hours (in 72.3% of the cases) (Kuznetsov and Tank 1966). Succinate dehydrogenase activity rise by cystamine in vitro in lymphocytes is indicator the development of acute cellular hypoxia (Vasin et al. 1999).

Cellular hypoxia under the influence of aminothiols in combination with the growth of the level of thiol groups in the cell due to radioprotector molecules and endogenous sources is an important components in the mechanism of radioprotective effect of sulfur-containing radioprotectors. The quota of acute hypoxia in protective effect of amifostine can consist 20–30% (Yuhas et al. 1973; Allalunis-Turner et al. 1989, Allalunis-Turner 1990).

**Figure 4:**
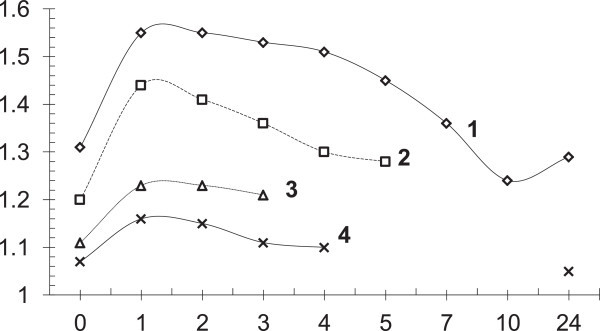
**Potentiation of radiation protective effect of cystamine administered IP to mice twice before exposure to radiation at various interval between two injection (Vasin and Antipov** 1972 **).** Abscissa: the time interval between administration of two doses of cystamine, hours; value 0 stands for a single administration of the drug. Ordinate: radiation protective effectiveness of cystamine, DRF: 1 - 150 mg/kg, 2 - 100 mg/kg, 3 - 75 mg/kg, 4 - 50 mg/kg. Note: cystamine was administered intraperitoneally, with the second dose injected 5-10 minutes prior to γ-irradiation.

It seemed likely “biochemical shock” induced aminothiols over five-six hours can potentiate radioprotective effect at repeated their administration with the interval of some hours. As shown in Figure 4, repeated IP injection of cystamine in the dose 150 mg/kg over hours five increase twice an efficacy of radioprotectors in the term of DRF. In case of decrease of the dose of cystamine, the time of the potentiation shortens up to two-three hours.

However, long time everyday administration of aminothiols in high doses causes decrease of their protective efficacy through the cumulation of their cytostatic or cytotoxic effects inhibited early post-radiation reparation of radiosensitive tissues (Travis et al. 1988; Coia and Brown 1989; Vasin et al. 1991, Vasin et al. 2004b).

Amifostine currently finds its application in clinical practice as a radioprotectant and chemoprotector during the radio-chemotherapy of patients with head and neck tumors, lung cancer and breast cancer, allowing the reduction of the radiotoxicity and toxicity of cytotoxic preparations (Trog et al. 1999; Feng et al. 2012; Koukourakis et al. 2013).

According to my calculation of clinic data by Trog et al. (1999) on the reduction of the symptoms of post-radiation mucositis during the radiotherapy treatment of head and neck cancer patients, DMF for amifostine is equal to 1.37 (Vasin 2014a).

*The role of pharmacological hypoxia in radiosensitive tissues in the mechanism of the action of radioprotectors from the family of vasoactive biogenic amine and their derivatives*

Endogenous vasoactive neuromediators such as adrenaline, noradrenaline, dopamine, serotonin, histamine, acetylcholine, prostaglandins, and purine nucleotides induce their radioprotective action through cell receptor mechanism by developing of acute circulatory and/or cellular hypoxia in radiosensitive tissues.

The first information about the ability of biogenic amines having a pronounced radiation protective effect to reduce the oxygen content in the hematopoietic tissues allowed van der Meer and van Bekkum (1959, 1961) and Konstantinova and Graevskii (1960) to propose a hypothesis about the role of pharmacological hypoxia as the main mechanism of the radiation protective action of these compounds. This idea was also supported by the experimental data on the reduction of radiation protective properties of biogenic amines after the application of pharmacological antagonists which block their effects on blood vessels, as well as under the influence of hyperoxia partially eliminating the disturbances in the oxygen delivery to tissues in the case of hemo-circulatory disorders under the action of biogenic amines (Van den Brenk and Moore 1959; van den Brenk and Haas 1961; van den Brenk and Jamieson 1962).

The role of pharmacologically induced disturbances of the local blood flow in hemopoietic tissues in the implementation of radiation protective action for many derivatives of serotonin at different doses and methods of application was confirmed later (Zherebchenko and Suvorov 1963; Hasegawa and Landahl 1967; Iarmonenko et al. 1970; Prewitt and Musacchia 1975; Vasin et al. 1984, 1987). Similar correlation for sympathomimetics was not always quite explicit (Kulinskii and Zolochevskaia 1973). Nevertheless, application of pharmacological antagonists eliminated the radioprotective effect epinephrine, norepinephrine, phenylephrine, and indralin (Kulinskii et al. 1986; Vasin et al. 1996, 2001). The same effect was observed with animal radiation exposure in an atmosphere of increased oxygen pressure (van den Brenk and Haas 1961; van den Brenk and Jamieson 1962; Vasin et al. 1979, 1996, Vasin et al. 1997a).

The real opportunities for the modification of the radiosensitivity of the organism in the case of pharmacologically induced disorders of the oxygen delivery to tissues can be compared with the radiation protective effect of the hypoxic hypoxia under the conditions of the drop of oxygen tension in the blood as low as twice or three times. The increase of radioresistance of animals breathed by gaseous hypoxic mixture (GHM) during irradiation was first of all obtained by Dowdy et al. (1950).

**Figure 5:**
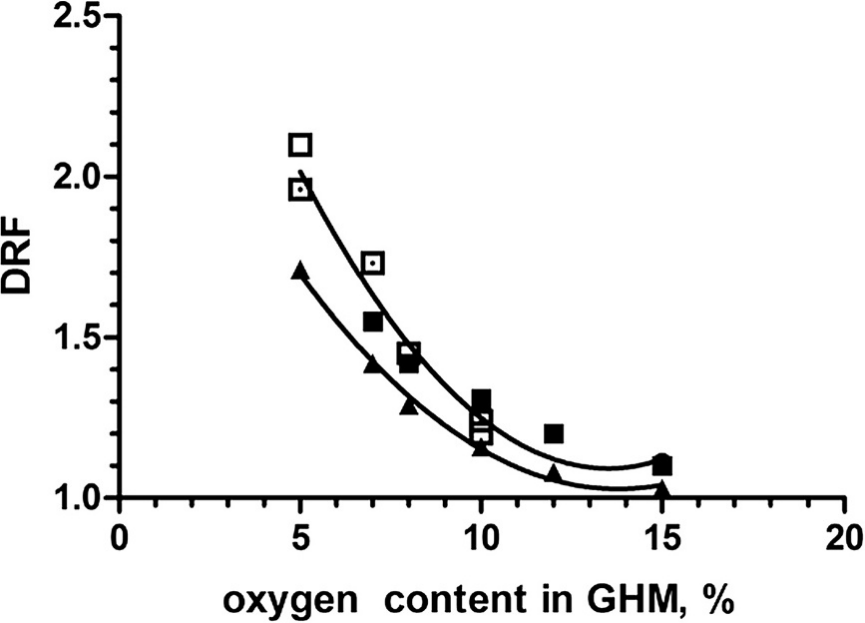
**Radioprotective efficacy of acute hypoxic hypoxia at respiration by gaseous hypoxic mixture with various content of oxygen during irradiation of mice and rats.** Abscissa: oxygen content in gaseous hypoxic mixture, %; Ordinate: radioprotective efficacy of GHM, DRF, Note: squares – mice, triangle – rats, black squares and triangle – (Vasin 1986), white squares – (Hasegawa and Landahl 1967), white square with point – (Iarmonenko et al. 1980).

Figure 5 shows radioprotective effect of normobaric hypoxic hypoxia with respiration of GHM with the deficient oxygen content from 15% to 5% in the experiment on mice and rats. Reduction of the oxygen delivery to tissues can lead to the shifts in the radioresistance of the organism close to the theoretically possible for the oxygen effect.

Oxygen tension level in cells with the acute reduction of the oxygen delivery to tissues is mainly determined by the intensity of the tissue respiration, which, for example, in dogs and rats is 5 and 2 times as low as that in mice that, in all probability, is a cause for the reduction of the radiation protective action of GHM in these animal species (Vasin 1986, Vasin et al. 1997a).

As shown in Figure 5, the effect of GHM in the experiment on rats is lower than that in mice (Vasin 1986). In the experiments on large animals (dogs) the radiation protective action of GHM was even less pronounced and did not exceed 50-60%, which is not higher than 1.2-1.3 in terms of DRF (Strelkov et al. 1974; Titov et al. 1977; Vasin et al. 1997a)*.*

Reduction of the radiation protective properties of mexamine, an active derivative of serotonin, in the experiments on rats and dogs, as compared with the effect on mice is associated, as in the case of application of hypoxic mixtures, with the influence of a lower initial level of the tissue respiration (Vasin 1986; Vasin et al. 1997a).

A high radioprotective efficiency of indralin in the large animals (dogs, monkeys) (Vasin et al. 1997b, 2014a) is largely predetermined, in addition to the vasoconstrictive effect, by its ability as a α_1_-adrenomimetic to stimulate the oxygen consumption in hematopoietic tissues (Vasin et al. 2013) thus inducing acute cellular hypoxia (Vasin et al. 1997a, 1999).

Besides, biogenic amines inducing radioprotective effect at their administration before irradiation have radiation mitigating properties when they are applied after exposure to radiation. Rixon and Baird (1968) showed first of all mitigatory effect of serotonin at repeated its administration after irradiation. Later, mitigating properties were observed when mexamine (5-methoxitryptamine) (Shashkov et al. 1971), adrenaline (Smirnova et al. 1984a) or α_1_-adrenomimetic indralin were injected at once after irradiation (Vasin et al. 2008b, 2014b). The mitigatory effect of indralin achieved steep rise in the condition of partial shielding of body (Vasin et al. 2008a). Radiation mitigatory effect of serotonin was detected in vitro (Smirnova et al. 1984b).

As noted, the sympathetic nervous system is actively involved in the regulation of the mobilization and migration of haematopoietic cells (Katayama et al. 2006). This characteristic feature accounts for the fact that norepinephrine (Maestroni et al. 1997) and α_1_-adrenomimetic indralin (Vasin et al. 2006, Vasin 2012) reduce the hemotoxicity of carboplatin. Catecholamines also enhance the stress-associated increase in the secretion of pro-inflammatory cytokines interleukin (IL)-1 and IL-6, which contribute to the mobilization of myeloid hematopoiesis (Johnson et al. 2005). The mechanism of hematopoiesis stimulation is probably associated with an activation of tissue transglutaminase and new G-protein-coupling α1-adrenergic receptors (Feng et al. 1999; Zhang et al. 1999; Park et al. 2001) that are able to activate nuclear factor (NF)-kappa B (Chen and Minneman 2005; Kumar and Mehta 2012). This is associated with a subsequent increase in gene expression of pro-inflammatory cytokines, including IL-1, tumour growth factor, IL-6, and IL-12, which promote cell proliferation of myeloid hematopoiesis (Zhao et al. 2012).

This study found that the mitigating effects of indralin, when it is administered after exposure to supralethal dose of radiation and when it is administered together with shielding of the abdominal region of rats, can not be explained by hypoxic mechanisms of α1-adrenomimetic activity. Radioprotectors cannot neutralize the “oxygen effect” when they are admin-istered after radiation exposure. The mitigable effect of radioprotectors applied after irradiation on the haematopoietic tissue is implemented through their interference with the processes of accelerated proliferation and migration of stem cells and progenitor cells of the myeloid group from the haematopoietic tissues that are protected by partial shielding.

Experimental principles for the use of hypoxia in radiation therapy have been presented (Jarmonenko et al. 1975). There are first clinic investigations of radioprotective effect of radioprotector mexamine (Voĭtkevich and Palyga 1974) and GHM (Strelkov et al. 1985; Goldobenko et al. 1995). Mexamine as a mitigator is used to reduce chemotoxicity of chemotherapy (Lissoni et al. 2003, 2012; Lissoni 2007). Indralin (B-190) is used as a radioprotective agent for medical protection of personnel during emergency situations at nuclear power plants (Ilyin et al. 1994, 2012, 2013).

***Radiomitigators (oestrogens, androgens, cytokines, and immunological adjuvants etc) that exert their effect on system levels by promoting the acceleration of the post‐radiation restoration of radiosensitive tissues through an activation of pro-inflammatory signalling pathways and a stimulation of HSPC and MSC mobilization***

Second group of radioprotective agents using in medicine exert their effect on system levels by promoting the acceleration of the post‐radiation restoration of radiosensitive tissues, and is not directly connected with the primary radiation-chemical and biochemical processes in cells that occur during the absorption of the energy from ionizing radiation. For this reason, these agents are effective not only at administration before irradiation, but also during the early period after, acting as mitigators. Their DRF is not restricted by an “oxygen effect”, but is limited by the physiologic actions of systems of the body, through which their pharmacological properties are mediated. For example according to available data (Lukashin and Sofronov 1996), the greatest radiation protective effect of the agent from the given group heparin takes place in the radiosensitive strains of CBA mice (DRF = 1.2), while it is almost completely absent in radioresistant animals C57Bl/6.

In some cases these agents have protective effects that are similar to the action of “classic” radioprotectors. However, their dependence on the functions of complex systems is reduced, so that most of the radiation protection takes place in radiation injuries of hematopoietic tissues, although it is seen in other tissues (e.g., intestines, skin) with acute radiation syndrome (ARS) (Smith et al. 1958, Ainsworth and Mitchell 1968, Monette et al. 1972, Wu and Miyamoto 1990, Roberts et al. 1993).

Given agents can exert their effects through activation of the reticuloendothelial system. They increase phagocytic function of mononuclear cells in the blood and tissues, stimulate the migration of hematopoietic cells and antibody formation, and increase resistance to exogenous infections (Lukashin and Morozova 1981; Klestova et al. 1984). Their radiation protective effects are related to their ability to accelerate the processes of post-radiation regeneration of the hematopoietic system and, above all, to restore myelopoiesis (Neta et al. 1986, Neta and Oppenheim 1988).

The common feature of these radiation protective agents is that the development of an elevated radioresistance due to these agents requires a certain period of time, usually hours or days. The optimum radiation protective effect exerted by long-acting radioprotectors is observed when they are used within a period of several hours to 3 to 4 days before exposure to radiation at doses not exceeding the levels that can result in bone marrow toxicity of ARS. These agents maintain their effectiveness for up to 2 weeks. A long-term action is not reflected in their tolerability because it is not closely related to their pharmacokinetics. These agents are not effective after exposure to radiation at super-lethal doses, distinguishing them from “classic” radioprotectors (Neta et al. 1995)

The radiation protective agents exerting their effects through second pathway described above include: (I) hormones or steroid structure and synthetic analogs, such as estrogens and androgens; (II) immunologic adjuvants and high-molecular structures of microbe, plant, and animal sources (vaccines, endotoxins, polysaccharides, glucans, polynucleotides); (III) cytokines such as inflammatory interleukins (IL-1beta, IL-8, IL-12, and IL-18), tumor necrosis factors (TNF), hemopoietic growth factors (G-CSF, GM-CSF, M-CSF, IL-3), soluble cytokines — interferons, and (IV) immune regulatory peptides.

The common feature for their action is the activation of the transcription factor NF-kB separately as well as together with AP-1 и STAT-3. This leads to the expression of genes responsible for cell proliferation, reduction of apoptosis of stem cells, and an increase in their survival rate after radiation damage. Immune defense is also improved, through an increased production of pro-inflammatory cytokines and hematopoietic growth factors (Shannon et al. 1997; Aggarwal et al. 2004; Chung et al. 2006b; Park et al. 2001). NF-kB, a "rapid-acting" primary transcription factor, is in a non-active state in the cytosol and is activated through IL-1beta, TNF, and lipopolysaccharide (LPS) receptors, bacterial and viral antigens, and by reactive oxygen species (ROS) and ionizing radiation (Qin et al. 2005; Gilmore 2006; Hayden and Ghosh 2011; Gerondakis et al. 2012). The agents of this group act as radiomitigators, and when administrated early after irradiation (up until clinic signs of ARS are apparent) their ability for myelopoiesis stimulation is preserved. This effect is of interest for use of these agents for emergency and early treatment of ARS (Neta 1988; MacVittie et al. 2005; Drouet et al. 2008). From this group of preparations, GM-CSF, G-CSF, IL-1, IL-3, and IL-2 are used in clinical practice as mitigators and therapeutic agents to minimize radio- and chemotoxicity (Robak et al. 1993; Baranov et al. 1994; Hartmann et al. 1997; Gershanovich et al. 2001, Westermann et al. 2001).

NF-kB activation is also possible through G-protein-coupled receptor (GPCR) mediators (biogenic amines, nucleosides, prostanoids, and angiotensin) and hormone nuclear receptors (Papayannopoulou et al. 2003, Katayama et al. 2006). These compounds also have the ability as mitigators to exert a favorable effect on the post-radiation recovery of hematopoiesis (Lebedev et al. 1994, Haznedaroglu and Oztürk 2003, Watanabe et al. 2006, Vasin et al. 2008a, Gudkov et al. 2009).

***Radiomodulators (natural antioxidants, anti-inflammatory agents, inhibitors of angiotensin converting enzymes etc) that increase the resistance of the body to irradiation and other unfavourable environmental factors by adaptively shifting the effectiveness of the antioxidative protection of the organism***

The mechanism of the radioprotective action of third group of agents is worthy of a detailed analysis in view of their great biological importance. The third group of radiation protective agents includes drugs and nutritional supplements that can elevate the resistance of the organism to adverse environmental factors, including exposure to ionization by means of modulating the gene expression through a hormetic effect of small doses of stressors and a “substrate” maintenance of adaptive changes, resulting in an increased antioxidant protection of the organism. Recently, they belonged to “biological protection” agents or stimulators of radioresistance (Saksonov 1975, Vasin 1999, Nair et al. 2001). Now, the third group of agents has a reason to refer to radiomodulators as original class of radioprotective agents. By definition, radiomodulators have both radiation protective features (relative to normal healthy tissues) and radiosensitizing properties towards cancer cells (Arora et al. 2008). Unfortunately, radiomodulators are not a member of present-day classification of radiation protective agents (Stone et al. 2004).

Radiomodulators have anti-inflammatory, anti-bacterial, anti-oxidant, and anti-mutagenic properties (Table 1) (Fang et al. 2002; Hong et al. 2006; Izzi et al. 2012). Similar properties are manifested in natural antioxidants. These agents, acting through the same mechanism may have a beneficial effect when administered before, during, and immediately after exposure to radiation; they also have a therapeutic effect in the development of clinical symptoms of radiation injury (Landauer et al. 2003; Landauer 2008; Arora et al. 2008).

**Table 1:** **The mechanisms and optimal conditions for radioprotective action of flavonoids (Vasin** 2014b **)**

Pharmacological action	The mechanisms of anti-radiation action	The optimal condition of protective action
Antioxidan	Enzyme and non-enzyme anti-radical effect, activation of Nrf2/KeapI and Sirtuin/FoxO pathway	Low-dose-rate irradiation, repeated prophylactic dose of drugs and nutrient supplement
Pro-oxidant	Activation the NF-κB pathway, increase of pro-inflammatory cytokines → stimulant effect of myelopoiesis	High-dose-rate and lethal irradiation, ovendose of drugs, delayed protective effect
Estrogenic	Immunostimulant action, G-CSF increase → stimulant effect of myelopoiesis	High-, low-dose-rate, and lethal, non-lethal irradiation, therapeutic dose of drugs, delayed protective effect
Anti- carcinogenic	Inhibition the NF-κB pathway, and the mutation or hyper- expression of the Nrf2-Keap1 signaling pathway in tumor genesis, and angiogenesis	Low-dose-rate irradiation, repeated prophylactic dose of drugs and nutrient supplement
Anti-inflammatory	Inhibition the NF-κB pathway, pro-inflammatory cytokine decrease → mitigation of acute radiation syndrome, long-term radiation oxidative stress and post-radiation fibrosis of tissues	High-, low-dose-rate, lethal and non-lethal irradiation, therapeutic dose of drugs, delayed protective effect

These agents can enhance resistance of the body to environmental factors that are adverse for human health, including ionizing radiation, with reducing the risk of carcinogenic effects and decreasing the biological age (Goel and Aggarwal 2010; Epperly et al. 2011; Lee et al. 2013). The best practical value of this medications consists, above all, in the fact that they can be considered as the agents for prophylaxis against the development of oxidative stress with long-term (months, years), low-rate-dose ionizing irradiation (Turner et al. 2002, Prasad 2005). These agents were previously and are currently being developed for use during long-term, low-rate-dose exposures to radiation, under conditions of long space missions (Chertkov and Petrov 1993; Rabin et al. 2005; Ushakov and Vasin 2011). Since these substances have low toxicity and do not have side effects at the administered doses, they can be used repeatedly, lifelong if necessary, with alterations in the cycles of metabolic correction and substrate therapy depending on the appearance of symptoms of chronic oxidative stress.

The theoretical basis for the research into the mechanism of increasing the nonspecific resistance of the organism under the influence of pharmacological agents is from a classic study by *Selye* (1985) and from additional research in this area, including studies investigating into the formation of different adaptive stages, depending on the strength and duration of the impact of adverse environmental factors (Garkavi et al. 1980; Lindsay 2005; Speciale et al. 2011).

The fundamentally important characteristic feature of the pharmacological agents that enhance the nonspecific resistance of the organism is the fact that their optimal effect can be formed due to the slowly proceeding structural signs manifesting themselves as the alteration and complication of the adaptive phases of “activation” and “training” supported by the definite rhythm of repeated exposures to low doses of an adaptogen (Garkavi et al. 1980).

Acting as low-dose stressors through a hormetic mechanism and a “substrate” support of adaptive shifts radiomodulators results in an increase in the antioxidant defense of the body and the rearrangement of its functioning in the new environment with the modulation of gene expression of antioxidant response elements by activation of NRF2/KEAPI and SIRTUIN/FOXO pathways and a decrease in the transcription factor NF-kB (Renard et al. 1997; Chen et al. 2005; Paur et al. 2010). According to their action, these agents are to some extent opposite to drugs of noted above second group—classical mitigators (e.g., vaccines, LPS, pro-inflammatory cytokines) which have pro-inflammatory properties and whose mechanism is based on the activation of the transcription factor NF-kB.

The repeated administration of vitamin complexes results in the increase of the total nonspecific resistance, including some degree of enhancement of the radiation resistance of the organism. This is associated with the maintenance of a specific tonus of the pituitary-adrenal system. For example, ascorbic acid (which is essential for the functioning of the adrenal glands where its concentration is sustained at a high level and exceeds that in other tissues) is a cofactor for the synthesis of adrenaline and glucocorticoids (Bornstein et al. 2003, Patak et al. 2004). The basis of the pathogenetic mechanism for their action lies in the stimulation of the neuro-immuno-endocrine system with the activation of individual axes of regulation (pituitary-adrenal, hypothalamic-thymic, and pituitary-thyroid), whose predominance depends on the nature, dose, and pattern of administration of a pharmaceutical agents. A direct substrate regulation of the antioxidant system and biosynthetic processes, which are important for the post-radiation reparation of the tissues of the body, is also possible when natural antioxidants are used as nutritional supplements—vitamins, nucleotides, oligopeptides, amino acids, and other compounds. However, in this case system reactions affecting the neuro-immuno-endocrine regulation in the organism are inevitable.

Dietary antioxidants such as vitamins A, C, and E, polyphenols, anthocyanins, flavonoids, isothiocyanates, and other natural antioxidants are considered as impacting redox reactions (Meyers et al. 2008). Those with high oxidant potential can directly interact with radiation-induced radicals; this has been recently considered as a basic mechanism of their radioprotective action. However, natural antioxidants, in all probability, participate in the gene regulation of the antioxidant system of the organism (Landauer 2008). Their indirect involvement as antioxidants can be achieved by means of inhibition of the redox-sensitive transcription factors (NF-kB, activator protein-1) and pro-oxidant enzymes (iNOS, cyclooxygenase, xanthine oxidase) through the induction of antioxidant enzymes (gluthation-5-transferase, superoxide dismutase), and phase II of cellular respiration (Frei and Higdon 2003). Experiments in vitro revealed that genistein and quercetin are able to significantly increase the gene promoter activity of glutathione peroxide and superoxide dismutase in the absence of the effect on the activity of catalase (Ullmann et al. 2008). Genistein also increased the expression of metallothionein genes (Chung et al. 2006a) and suppressed post-radiation pro-inflammatory cytokine induction (Grace et al. 2007, Singh et al. 2009, Ha et al. 2013). Quercetin inhibits the synthesis of iNOS through the inhibition of NF-kB and STAT1 (Meyers et al. 2008) and suppresses the growth of xenograft A2780S ovarian tumors by causing cancer cell apoptosis and inhibition of angiogenesis in vivo (Gao et al. 2012). Epigallocatechin-3-gallate protects cells from ionizing radiation via heme oxygenase-1 overexpression that suppresses ROS generation (Zhu et al. 2014).

Drugs with NF-κB pathway suppressive and anti-inflammatory actions, such as angiotensin-converting enzyme and prostaglandin biosynthesis inhibitors, genistein and melatonin, can mitigate post-radiation fibrosis of kidney, lungs, and skin (Table 1) (Sklobovskaia et al. 1984; Molteni et al. 2001; Vasin et al. 2004a; Day et al. 2008; Sieber et al. 2011; Medhora et al. 2012; Kma et al. 2012) and post-radiation myelosuppression of ARS (Davis et al. 2007, 2008; Day et al. 2013; Vasin et al. 2014c).

Anti-inflammatory effect of ginestein suppresses post-radiation increase of Il-1-beta, Il-6 and COX-2 in hematopoietic tissues that is regard as key mechanism of radioprotective action the isoflanone (Ha et al. 2013). Indeed, inhibition of COX-2 by meloxicam, administered shortly after irradiation increases survival of lethally irradiated mice (Hofer et al. 2011). Natural delta-tocotrienol significantly enhanced survival in total-body irradiated mice, through Erk activation-associated mTOR survival pathways via inhibition of radiation-induced production of pro-inflammatory factors interleukin-1β and Il-6 and suppressed expression of protein tyrosine kinase 6, a stress-induced kinase that promotes apoptosis in mouse intestinal cells (Li et al. 2013) (Table 1).

The favorable action of natural antioxidants in the presence of the chronic oxidative stress is the reduction in the intensity and time needed to implement the remote impact of radiation damage to particular tissues and systems, primarily associated with their important role in the functioning of the vascular system.

This prevents the development of atherosclerosis, disorders of the microcirculation in tissues and their fibrosis, and the manifestation of chronic inflammatory processes in the patients with the immune system disorders; in other words, premorbid states leading to, as noted above, cardiovascular and endocrine diseases and the progression of carcinogenesis (Zhao and Robbins 2009).

It is also known that the bioflavonoids epigallakatehin-3-gallate, luteolin, quercetin, kaempferol, apegenin, and taxifolin are able to block the fatty acid synthase, which is the basis for the anti-carcinogenic action of these compounds (Brusselmans et al. 2005).

The tissues of the body contain ascorbic acid at a certain concentration; cell membranes contain tocopherols. There is a recirculation of the oxidized and reduced forms of these natural antioxidants, which supports the antioxidant protection of cell membranes. It involves bioflavonoids, which are the most powerful antioxidants, and the antioxidant system of cells as a whole (Table 1). Quercetin is able to accumulate in mitochondria and bind to the DNA molecule (Janjua et al. 2009).

These are the reasons why the administration of natural antioxidants have a pathogenetic justification for exposures to chronic (months, years) low-rate-dose ionizing radiat (Okunieff et al. 2008). The reduction of the consequences of the exposure to the long-term radiation can be achieved by providing an adequately balanced diet rich in vitamins, fibers, and nutritious animal proteins.

The realization of the action of natural antioxidants takes a certain period of time, so the effect is enhanced following the repeated administration of vitamins (Orsolić et al. 2007; Benković et al. 2009). A peculiar feature of pharmacodynamics of the preparations comprising the "biological shield" is that the modifications in the radiation resistance of the organism under their impact do not happen immediately, but gradually over repeated administration, more often within 2 weeks, and can be maintained at the elevated level during the entire course of treatment and prevention. When the preventive measures are well tolerated, they can be repeated without any loss of their effectiveness (Table 1).

Antioxidants (quercetin, vitamins A and C, beta-carotene, selenium, melatonin, and glutathione) can be used in clinical practice for the decrease of the toxicity of radio-chemotherapy for cancer patients (Lamson and Brignall 2000, Vasin et al. 2004a, 2004b).

## Conclusion

For most of these agents, it is possible to distinguish three main pathways for the implementation of the mechanism of action, each of which have their own specificity in predetermining the pharmacodynamic properties and possible methods in their practical application: 1) radiation protective agents, with the implementation of radiation protective action taking place at the cellular level in the course of rapidly proceeding radiation-chemical reactions. At the same time, when the ionizing radiation energy is absorbed, these agents partially neutralize the “oxygen effect” as a radiobiological phenomenon, especially in the radiolysis of DNA; 2) radiation protective agents that exert their effect at the system level by accelerating the post-radiation recovery of radiosensitive tissues through activation of a number of pro-inflammatory signaling pathways and an increase in the secretion of hematopoietic growth factors, including their use as mitigators in the early period after irradiation prior to the clinical development of acute radiation syndrome (ARS). 3) radiomodulators including drugs and nutritional supplements that can elevate the resistance of the organism to adverse environmental factors, including exposure to ionization by means of modulating the gene expression through a hormetic effect of small doses of stressors and a “substrate” maintenance of adaptive changes, resulting in an increased antioxidant protection of the organism. Anti-inflammatory properties of radiomodulators can be key basis of realization of their radioprotective action.

The possibility that radiation protective agents may implement their action by various routes and through different mechanisms mentioned above cannot be excluded. For example, indralin, a radioprotector with urgent action can act as a mitigator (Ilyin et al. 1994; Vasin et al. 2008a, [b], 2014b), while the radioprotector amifostine is capable of stimulating the antioxidant system of the body through the activation of Mn-SOD (Grdina et al. 2009), and the radiomodulator α-tocopherol succinate, at very high doses, possibly acting as a pro-oxidant, induces G-CSF mobilization (Singh et al. 2010).

## Authors’ information

Professor of Department of Medicine of Catastrophe, Russian Medical Academy of Post-Graduated Education, Street Polikarpova 10, Moscow 125284, Russian Federation.

## References

[CR1] Aggarwal BB, Takada Y, Shishodia S, Gutierrez AM, Oommen OV, Ichikawa H, Baba Y, Kumar A (2004). Nuclear transcription factor NF-kappa B: role in biology and medicine. Indian J Exp Biol.

[CR2] Ainsworth EJ, Mitchell FA (1968). Increased survival of irradiated dogs given typhoid vaccine before or after irradiation. Radiat Res.

[CR3] Alexander P, Charlesby A (1954). Physico-chemical methods of protection against ionizing radiations.

[CR4] Allalunis-Turner MJ, Walden TJ, Sawich C (1989). Induction of mar-row hypoxia by radioprotective agents. Radiat Res.

[CR5] Allalunis-Turner MJ (1990). Reduced bone marrow pO2 following treatment with radioprotective drugs. Radiat Res.

[CR6] Arora R, Kumar R, Sharma A, Tripathi RP, Arora R (2008). Radiomodulatory compounds of herbal origin for new frontiers in medicine, homeland security, management of radiological incidents and space application. Herbal Radiomodulators: Applications in Medicine, Homeland Defense and Space.

[CR7] Baranov AE, Selidovkin GD, Butturini A, Gale RP (1994). Hematopoietic recovery after 10-Gy acute total body radiation. Blood.

[CR8] Benković V, Knezević AH, Dikić D, Lisicić D, Orsolić N, Basić I, Kopjar N (2009). Radioprotective effects of quercetin and ethanolic extract of propolis in gamma-irradiated mice. Arh Hig Rada Toksikol.

[CR9] Bornstein SR, Yoshida-Hiroi M, Sotiriou S, Levine M, Hartwig HG, Nussbaum RL, Eisenhofer G (2003). Impaired adrenal catecholamine system function in mice with deficiency of the ascorbic acid transporter (SVCT2). FASEB J.

[CR10] Brusselmans K, Vrolix R, Verhoeven G, Swinnen JV (2005). Induction of cancer cell apoptosis by flavonoids is associated with their ability to inhibit fatty acid synthase activity. J Biol Chem.

[CR11] Chen JC, Ho FM, Chao P-DL, Chen CP, Jeng KC, Hsu HB, Lee ST, Wen Tung W, Lin WW (2005). Inhibition of iNOS gene expression by quercetin is mediated by the inhibition of IkappaB kinase, nuclear factor-kappa B and STAT1, and depends on heme oxygenase-1 induction in mouse BV-2 microglia. Eur J Pharmacol.

[CR12] Chen ZJ, Minneman KP (2005). Recent progress in alpha1-adrenergic receptor research. Acta Pharmacol Sin.

[CR13] Chertkov KS, Petrov VM (1993). Pharmacological-chemical protection and substitutive therapy as component of system of radiation safety for cosmonauts in mission to Mars. Aviakosm Ekol Med.

[CR14] Chigareva NG, Morozova IN, Deev SP (1990). The mechanism of the radioprotective action of cystamine and mexamine. Radiobiologiia.

[CR15] Chung MJ, Kang AY, Lee KM, Oh E, Jun HJ, Kim SY, Auh JH, Moon TW, Lee SJ, Park KH (2006). Water-soluble genistin glycoside isoflavones up-regulate antioxidant metallothionein expression and scavenge free radicals. J Agric Food Chem.

[CR16] Chung YJ, Park BB, Kang YJ, Kim TM, Eaves CJ, Oh IH (2006). Unique effects of Stat3 on the early phase of hematopoietic stem cell regeneration. Blood.

[CR17] Coia LR, Brown DQ (1989). Protection on bone marrow by WR-2721 after fractionated irradiation. Int J Radiat Oncol Biol Phys.

[CR18] Davis TA, Clarke TK, Mog SR, Landauer MR (2007). Subcutaneous administration of genistein prior to lethal irradiation supports multilineage, hematopoietic progenitor cell recovery and survival. Int J Radiat Biology.

[CR19] Davis TA, Mungunsukh O, Zins S, Day RM, Landauer MR (2008). Genistein induces radioprotection by hematopoietic stem cell.

[CR20] Day RM, Barshishat-Kupper M, Mog SR, McCart EA, Prasanna PGS, Davis TA, Landauer MR (2008). Genistein protects against biomarkers of delayed lung sequelae in mice surviving high dose total body irradiation. J Radiat Res.

[CR21] Day R, Davis TA, Barshishat-Kupper M, McCart EA, Tipton AJ, Landauer MR (2013). Enhanced hematopoietic protection from radiation by the combination of genistein and captopril. Int Immunopharmacol.

[CR22] Doherty DG, Hollaender A (1960). Chemical protection to mammals against ionizing radiation. Radiation Protection and Recovery.

[CR23] Dowdy AH, Bennett LR, Chastain SM (1950). Protective action of anoxia against total body roentgen irradiation of mammals. Radiology.

[CR24] Drouet M, Delaunay C, Grenier N, Garrigou P, Mayol JF, Hérodin F (2008). Cytokines in combination to treat radiation-induced myelosuppresssion: evaluation of SCF + glycosylated EPO + pegylated G-CSF as an emergency treatment in highly irradiated monkeys. Haematol.

[CR25] Duplan JF, Fuhrer J (1966). Estimation of the radioprotective effect of aminoethylisothiouronium (AET) by enumeration of the splenic nodules. C R Séances Soc Biol Filiales.

[CR26] Epperly MW, Wang H, Jones JA, Dixon T, Montesinos CA, Greenberger JS (2011). Antioxidant-chemoprevention diet ameliorates late effects of total-body irradiation and supplements radioprotection by MnSOD-plasmid liposome administration. Radiat Res.

[CR27] Fahey RC (1988). Protection of DNA by thiols. Pharmacol Ther.

[CR28] Fang YZ, Yang S, Wu G (2002). Free radicals, antioxidants, and nutrition. Nutr.

[CR29] Feng M, Smith DE, Normolle DP, Knol JA, Pan CC, Ben-Josef E, Lu Z, Feng MR, Chen J, Ensminger W, Lawrence TS (2012). A phase I clinical and pharmacology study using amifostine as a radioprotector in dose-escalated whole liver radiation therapy. Int J Radiat Oncol Biol Phys.

[CR30] Feng JF, Gray CD, Im MJ (1999). Alpha 1B-adrenoceptor interacts with multiple sites of transglutaminase II: characteristics of the interaction in binding and activation. Biochemistry.

[CR31] Firket H, Lelievre P (1966). Effect de la cystamine sur la respiration, la phosphorylation oxydative et l`ultrastructure des mitochondries. Int J Radiat Biol.

[CR32] Frei B, Higdon JV (2003). Antioxidant activity of tea polyphenols in vivo: evidence from animal studies. J Nutr.

[CR33] Gao X, Wang B, Wei X, Men K, Zheng F, Zhou Y, Zheng Y, Gou M, Huang M, Guo G, Huang N, Qian Z, Wei Y (2012). Anticancer effect and mechanism of polymer micelle-encapsulated quercetin on ovarian cancer. Nanoscale.

[CR34] Garkavi LK, Kvakina EB, Mulatova AK, Shikhliarova AI (1980). Enhancement of antitumor resistance by means of small doses of adrenaline. Vopr Onkol.

[CR35] Gerondakis S, Banerjee A, Grigoriadis G, Vasanthakumar A, Gugasyan R, Sidwell T, Grumont RJ (2012). NF-κB subunit specificity in hemopoiesis. Immunol Rev.

[CR36] Gershanovich ML, Filatova LV, Ketlinsky SA, Simbirtsev AS (2001). Recombinant human interleukin-1 beta: new possibilities for the prophylaxis and correction of toxic myelodepression in patients with malignant tumors. II. Phase II study of the protective effect of recombinant human interleukin-1 beta on myelodepression induced by chemotherapy in cancer patients. Eur Cytokine Netw.

[CR37] Gilmore TD (2006). Introduction to NF-kB: players, pathways, perspectives. Oncogene.

[CR38] Goel A, Aggarwal BB (2010). Curcumin, the golden spice from indian saffron, is a chemosensitizer and radiosensitizer for tumors and chemoprotector and radioprotector for normal organs. Nutr Cancer.

[CR39] Goldobenko GV, Prorokov VV, Knysh VI, Barkanov AI: **Preoperative hypoxia-radiotherapy in multimodal treatment of patients with stage III colonic cancer].** *Vestnik Ross Akad Med Nauk* 1995, (4)**:**50–52. (in Russian) 7780343

[CR40] Grace MB, Blakely WB, Landauer MR (2007). Genistein-induced alterations of radiation-responsive gene expression. Radiat Measurements.

[CR41] Grdina DJ, Murley JS, Kataoka Y, Baker KL, Kunnavakkam R, Coleman MC, Spitz DR (2009). Amifostine induces antioxidant enzymatic activities in normal tissues and a transplantable tumor that can affect radiation response. Int J Radiat Oncol Biol Phys.

[CR42] Gudkov SV, Gudkova OY, Chernikov AV, Bruskov VI (2009). Protection of mice against X-ray injuries by the post-irradiation administration of guanosine and inosine. Int J Radiat Biol.

[CR43] Ha CT, Li XH, Fu D, Xiao M, Landauer MR (2013). Genistein nanoparticles protect mouse hematopoietic system and prevent proinflammatory factors after gamma radiation. Radiat Res.

[CR44] Hall EJ, Giaccia AJ (2012). Radiobiology for the Radiologist.

[CR45] Hartmann LC, Tschetter LK, Habermann TM, Ebbert LP, Johnson PS, Mailliard JA, Levitt R, Suman VJ, Witzig TE, Wieand HS, Miller LL, Moertel CG (1997). Granulocyte colony-stimulating factor in severe chemotherapy-induced afebrile neutropenia. N Engl J Med.

[CR46] Hasegawa AT, Landahl HD (1967). Studies on spleen oxygen tension and radioprotection in mice with hypoxia, serotonin and p-aminopropiophenone. Radiat Res.

[CR47] Hasegawa AT, Landahl HD (1970). Dose-redaction factor for radiation lethality in mice as function of dose of mercaptoethylamine. Radiat Res.

[CR48] Hayden MS, Ghosh S (2011). NF-κB in immunobiology. Cell Res.

[CR49] Haznedaroglu IC, Oztürk MA (2003). Towards the understanding of the local hematopoietic bone marrow renin-angiotensin system. Int J Biochem Cell Biol.

[CR50] Held KD (1988). Models for thiol protection of DNA in cells. Pharmacol Ther.

[CR51] Hofer M, Pospisil M, Dusek L, Hoferova Z, Weiterova L (2011). A single dose of an inhibitor of cyclooxygenase 2, meloxicam, administered shortly after irradiation increases survival of lethally irradiated mice. Radiat Res.

[CR52] Hong H, Foriska M, Landauer MR, Ledney GD (2006). Antibacterial activity of the soy isoflavone genistein. J Basic Microbiol.

[CR53] Hutchinson F (1961). Sulfhydryl groups and the oxygen effect on irradiated dilute solutions of enzymes and nucleic acids. Radiat Res.

[CR54] Jarmonenko SP, Neumeister K, Vainson AA, Krimker VM, Smakova NL, Petrosjan EP, Oelssner W, Melhorn H, Koch F, Arnold P, Johannsen U, Jahns J, Kamprad F (1975). Experimental principles for the use of hypoxia in radiation therapy. Radiobiol Radiother (Berl).

[CR55] Iarmonenko SP, Rampan II, Karochkin BB, Berezhnova LI, Ovakimov VG (1970). [Oxygen tension kinetics in critical organs under the effects of mexamine in comparison with its radiation-protective effect]. Radiobiologiia.

[CR56] Iarmonenko SP (1961). [Influence of cystamine to tissue respiration]. Radiobiologiia.

[CR57] Iarmonenko SP, Vainson AA, Magdon E (1980). [Oxygen effect and radiotherapy of tumours].

[CR58] Ilyin LA, Rudnyi NM, Suvorov NN, Chernov GA, Antipov BB, Vasin MV, Davydov BI, Mikhailov PP (1994). [Indralin – radioprotector of urgent action: radiation-protective property, pharmacology, mechanism of action, clinics].

[CR59] Ilyin LA, Ushakov IB, Vasin MV (2012). Radioprotective drugs in the system of radiation protection of exposed radiation workers and population in the case of radiation accidents. Med Radiol Radiat Safety.

[CR60] Ilyin LA, Ushakov IB, Vasin MV (2013). Radioprotective drugs in the system of radiation protection of exposed radiation workers and population in the case of nuclear accidents, The IRPA13 Abstract Book.

[CR61] Izzi V, Masuelli L, Tresoldi I, Sacchetti P, Modesti A, Galvano F, Bei R (2012). The effects of dietary flavonoids on the regulation of redox inflammatory networks. Front Biosc.

[CR62] Janjua NK, Siddiqa A, Yaqub A, Sabahat S, Qureshi R, ul Haque S (2009). Spectrophotometric analysis of flavonoid-DNA binding interactions at physiological conditions. Mol Biomol Spectrosc.

[CR63] Jeitner TM, Delikatny EJ, Ahlqvist J, Capper H, Cooper AJ (2005). Mechanism for the inhibition of transglutaminase 2 by cystamine. Biochem Pharmacol.

[CR64] Jellum E (1965). Interaction of cysteamine and cystamine derivatives with nucleic acids and nucleoproteins. Int J Radiat Biol.

[CR65] Johnson JD, Campisi J, Sharkey CM, Kennedy SL, Nickerson M, Greenwood BN, Fleshner M (2005). Catecholamines mediate stress-induced increases in peripheral and central inflammatory cytokines. Neuroscience.

[CR66] Katayama Y, Battista M, Kao WM, Hidalgo A, Peired AJ, Thomas SA, Frenette PS (2006). Signals from the sympathetic nervous system regulate hematopoietic stem cell egress from bone marrow. Cell.

[CR67] Klestova OV, Riabukha AK, Shapiro NI, Strelin GS (1984). Effect of endotoxin on the migration of hematopoietic colony-forming cells and on the repopulation of hematopoietic organs in partially irradiated mice. Radiobiologiia.

[CR68] Kma L, Gao F, Fish BL, Moulder JE, Jacobs ER, Medhora M (2012). Angiotensin converting enzyme inhibitors mitigate collagen synthesis induced by a single dose of radiation to the whole thorax. J Radiat Res.

[CR69] Koch R (1967). Untersuchungen über einen biologischen Strahlenschutz. 80 Mitteilung: Über quantitative Beziehungen des chemischen Strahlenschutzes zu seinen Wirkungsmechanismus. Strahlentherapie.

[CR70] Konstantinova MM, Graevskii EI (1960). [Tissue hypoxia as the mechanism of radioprotective action of epinephrine, heroin and morphin]. Dokl Akad Nauk SSSR.

[CR71] Koukourakis MI, Panteliadou M, Abatzoglou IM, Sismanidou K, Sivridis E, Giatromanolaki A (2013). Postmastectomy hypofractionated and accelerated radiation therapy with (and without) subcutaneous amifostine cytoprotection. Int J Radiat Oncol Biol Phys.

[CR72] Kulinskii VI, Klimova AD, IashunskiI VG, Alpatova TV (1986). Mechanism of the radioprotective effect of catecholamine receptor agonists. Inclusion in the radioprotective effect of both subtypes of alpha-adrenoreceptors. Radiobiologiia.

[CR73] Kulinskii VI, Zolochevskaia LI (1973). The absence of correlation between the effects of sympathomimetics on blood flow in internal organs, oxygen tension, and the viability of irradiated animals. Radiobiologiia.

[CR74] Kumar S, Mehta K (2012). Tissue transglutaminase constitutively acti-vates HIF-1α promoter and nuclear factor-κB via a non-canonical pathway. PLoS One.

[CR75] Kuznetsov VI, Tank LI (1964). The change of oxygen consumption of erythrocytes by cystamine. Radiobiologiia.

[CR76] Kuznetsov VI, Tank LI (1966). Pharmacology and clinic application of aminothiols.

[CR77] Lamson DW, Brignall MS (2000). Antioxidants and cancer therapy II: quick reference guide. Altern Med Rev.

[CR78] Landauer MR, Srinivasan V, Seed TM (2003). Genistein treatment protects mice from ionizing radiation injury. J Applied Toxicol.

[CR79] Landauer MR, Arora R (2008). Radioprotection by the soy isoflavone genistein. Herbal Radiomodulators: Applications in Medicine, Homeland Defense and Space.

[CR80] Lebedev VG, Moroz BB, Vorotnikova TV, Deshevoi IB (1994). Mechanism of radioresistance of the hematopoietic system after treatment with diethylstilbestrol. Radiats Biol Radioecol.

[CR81] Lee JH, Khor TO, Shu L, Su ZY, Fuentes F, Kong AN (2013). Dietary phytochemicals and cancer prevention: Nrf2 signaling, epigenetics, and cell death mechanisms in blocking cancer initiation and progression. Pharmacol Ther.

[CR82] Lehninger A, Schneider M (1959). Mitochondrial swelling induced by glutathione. J Biophys Biochem Cytol.

[CR83] Lelievre P (1965). Action de la cysteamine sur la consommation d`oxygene et phosphorylation oxydetive couplee mitochondries de foie de rat. Int J Radiat Biol.

[CR84] Lesort M, Lee M, Tucholski J, Johnson VW (2003). Cystamine inhib-its caspase activity. J Biol Chem.

[CR85] Li XH, Ghosh SP, Ha CT, Fu D, Elliott TB, Bolduc DL, Villa V, Whitnall MH, Landauer MR, Xiao M (2013). Delta-tocotrienol protects mice from radiation-induced gastrointestinal injury. Radiat Res.

[CR86] Lindsay DG (2005). Nutrition, hormetic stress and health. Nutr Res Rev.

[CR87] Lissoni P, Malugani F, Bukovec R, Bordin V, Perego M, Mengo S, Ardizzoia A, Tancini G (2003). Reduction of cisplatin-induced anemia by the pineal indole 5-methoxytryptamine in metastatic lung cancer patients. Neuro Endocrinol Lett.

[CR88] Lissoni P, Messina G, Rovelli F (2012). Cancer as the main aging factor for humans: the fundamental role of 5-methoxy-tryptamine in reversal of cancer-induced aging processes in metabolic and immune reactions by non-melatonin pineal hormones. Curr Aging Sci.

[CR89] Lissoni P (2007). Biochemotherapy with immunomodulating pineal hormones other than melatonin: 5-methoxytryptamine as a new oncostatic pineal agent. Pathol Biol (Paris).

[CR90] Lukashin BP, Morozova IN (1981). Heparin stimulation of the migration capacity of hematopoietic cells in irradiated rodents. Radiobiologiia.

[CR91] Lukashin BP, Sofronov GA (1996). Radioprotective effect of cystamine and heparin in experiments on mice with varying tolerances. Bull Exp Biol Med.

[CR92] MacVittie TJ, Farese AM, Jackson W (2005). Defining the full therapeutic potential of recombinant growth factors in the post radiation-accident environment: the effect of supportive care plus administration of G-CSF. Health Phys.

[CR93] Maestroni GJ, Togni M, Covacci V (1997). Norepinephrine protects mice from acute lethal doses of carboplatin. Exp Hematol.

[CR94] Maisin JH, Lambert G, Mandart M, Maisin H (1953). Therapeutic action of glutathione and beta-mercaptoethylamine against a lethal dose of x-rays. Nature.

[CR95] Maisin J, Mandart M, Lambert G, Maisin H (1953). Curative action of beta-mercapto-ethylamine in the rat irradiated with the liver protected. C R Séances Soc Biol Filiales.

[CR96] Maisin JH, Matterin G, Fredman-Manduzio A, van den Porren L (1968). Reduction of short and longtern radiation lethality by mixtures of chemical protectors. Radiat Res.

[CR97] Medhora M, Gao F, Jacobs ER, Moulder JE (2012). Radiation damage to the lung: mitigation by angiotensin-converting enzyme (ACE) inhibitors. Respirology.

[CR98] Meyers KJ, Rudolf JL, Mitchell AE (2008). Influence of dietary quercetin on glutathione redox status in mice. J Agric Food Chem.

[CR99] Molteni A, Moulder JE, Cohen EP, Fish BL, Taylor JM, Veno PA, Wolfe LF, Ward WF (2001). Prevention of radiation-induced nephropathy and fibrosis in a model of bone marrow transplant by an angiotensin II receptor blocker. Exp Biol Med (Maywood).

[CR100] Monette FC, Morse BS, Howard D, Niskanen E, Stohlman F (1972). Hemopoietic stem cell proliferation and migration following Bordetella pertussis vaccine. Cell Tissue Kinetic.

[CR101] Murley JS, Kataoka Y, Baker KL, Diamond AM, Morgan WF, Grdina DJ (2007). Manganese superoxide dismutase (SOD2)-mediated delayed radioprotection induced by the free thiol form of amifostine and tumor necrosis factor alpha. Radiat Res.

[CR102] Murley JS, Grdina DJ (1995). The effect of cycloheximide and WR-1065 on repair processes: a mechanism for chemoprotection. Carcin-ogenesis.

[CR103] Murley JS, Kataoka Y, Weydert CJ, Oberley LW, Grdina DJ (2006). Delayed radioprotection by transcription factor kB-mediated induction of manganese superoxide dismutase in human microvascu-lar endothelial cells after exposure to the free radical scavenger WR1065. Free Radic Biol Med.

[CR104] Nair CK, Parida DK, Nomura T (2001). Radioprotectors in radiotherapy. Radiat Res.

[CR105] Neta R, Douches S, Oppenheim JJ (1986). Interleukin 1 is a radioprotector. J Immunol.

[CR106] Neta R, Oppenheim JJ (1988). Cytokines in therapy of radiation injury. Blood.

[CR107] Neta R, Stiefel SM, Ali N (1995). In lethally irradiated mice interleukin-12 protects bone marrow but sensitizes intestinal tract to damage from ionizing radiation. Ann NY Acad Sciences.

[CR108] Neta R (1988). Role of cytokines in radioprotection. Pharmacol Ther.

[CR109] Neubert D, Lehninger A (1962). The effect of thiols and disulfides on water uptake and extrusion by rat liver mitochondria. J Biol Chem.

[CR110] Newton GL, Aguilera JA, Ward JF, Fahey RC (1996). Binding of radioprotective thiols and disulfides in Chinese hamster V 79 cell nuclei. Radiat Res.

[CR111] North S, El-Ghissassi F, Pluquet O, Verhaegh G, Hainaut P (2000). The cytoprotective aminothiol WR1065 activates p21waf-1 and down regulates cell cycle progression through a p53-dependent pathway. Oncogene.

[CR112] Okunieff P, Swarts S, Keng P, Sun W, Wang W, Kim J, Yang S, Zhang H, Liu C, Williams JP, Huser AK, Zhang L (2008). Antioxidants reduce consequences of radiation exposure. Adv Exp Med Biol.

[CR113] Ormerod MG, Riesz P (1967). An unusual reaction of hydrogen sulphide and hydrogen iodide with free radicals in irradiated dry deoxyribonucleic acid. Biochim Biophys Acta.

[CR114] Orsolić N, Benković V, Horvat-Knezević A, Kopjar N, Kosalec I, Bakmaz M, Mihaljević Z, Bendelja K, Basić I (2007). Assessment by survival analysis of the radioprotective properties of propolis and polyphenolic compounds. Biol Pharm Bull.

[CR115] Papayannopoulou T, Priestley GV, Bonig H, Nakamoto B (2003). The role of G-protein signaling in hematopoietic stem/progenitor cell mobilization. Blood.

[CR116] Park H, Park ES, Lee HS, Yun HY, Kwon NS, Baek KJ (2001). Distinct characteristic of Galpha(h) (transglutaminase II) by compartment: GTPase and transglutaminase activities. Biochem Biophys Res Commun.

[CR117] Patak P, Willenberg HS, Bornstein SR (2004). Vitamin C is an important cofactor for both adrenal cortex and adrenal medulla. Endocr Res.

[CR118] Patt HM, Mayer S, Straube R, Jackson E (1953). Radiation dose reduction by cyseine. J Cellular Comparative Physiol.

[CR119] Paur I, Balstad TR, Kolberg M, Pedersen MK, Austenaa LM, Jacobs DR, Blomhoff R (2010). Extract of oregano? Coffe, thyme, clove, and walnuts inhibits NF-kappaB in monocytes and in transgenic reporter mice. Cancer Prev Res (Phila).

[CR120] Pluquet O, North S, Bhoumik A, Dimas K, Ronai Z, Hainaut P (2003). The cytoprotective aminothiol WR1065 activates p53 through a non-genotoxic signaling pathway involving c-Jun N-terminal kinase. J Biol Chem.

[CR121] Prasad KN (2005). Rationale for using multiple antioxidants in protecting humans against low doses of ionizing radiation. Br J Radiol.

[CR122] Praslicka M, Hill M, Novak L (1962). Protective action of 2,4-dinitrophenol against x-radiation injury. Radioprotective effect of 2,4-dinitrophenol. Int J Radiat Biol.

[CR123] Prewitt RL, Musacchia XJ (1975). Mechanisms of radio-protection by catecholamines in the hamster (Mesocricetus auratus). Int J Radiat Biol.

[CR124] Purdie JW, Inhaber ER, Schneider H, Labelle JL (1983). Interaction of cultured mammalian cells in the WR-2721 and its thiol WR-1065: implications for mechanisms of radioprotection. Int J Radiat Biol.

[CR125] Qin H, Wilson CA, Lee SJ, Zhao X, Benveniste EN (2005). LPS induces CD40 gene expression through the activation of NF-kB and Stat-1alpha in macrophages and microglia. Blood.

[CR126] Rabin BM, Shukitt-Hale B, Joseph J, Todd P (2005). Diet as a factor in behavioral radiation protection following exposure to heavy particles. Gravit Space Biol Bull.

[CR127] Ramdas J, Warrier RP, Scher C, Larussa V (2003). Effects of ami-fostine on clonogenic mesenchymal progenitors and hematopoietic progenitors exposed to radiation. Pediat Hematol Oncol.

[CR128] Renard P, Zachary MD, Bougelet C, Mirault ME, Haegeman G, Remacle J, Raes M (1997). Effect of antioxidant enzyme modulations on interleukin-1-induced nuclear factor kappa B activation. Biochem Pharmacol.

[CR129] Rixon EH, Baird KM (1968). The therapeutic effect of serotonin on the survival of x-irradiated rats. Radiat Res.

[CR130] Robak T, Krykowski E, Warzocha K, Kończalik P (1993). Application of hematopoietic growth factors (G-CSF and GM-CSF) in the treatment of chemotherapy-induced or idiopathic bone marrow failure. Acta Haematol Pol.

[CR131] Roberts DB, Travis EL, Tucker SL (1993). Interleukin-1 dose, mouse strain, and end point as they affect protection of mouse jejunum. Radiat Res.

[CR132] Romano MF, Lamberti A, Bisogni R, Garbi C, Pagnano AM, Auletta P, Tassone P, Turco MC, Venuta S (1999). Amifostine inhibits hematopoietic progenitor cell apoptosis by activating NF-kappaB/Rel transcription factors. Blood.

[CR133] Romashko OO, Lebedev VG, Moroz BB (1990). [The role of the hemopoietic microenvironment on the hemopoiesis–stimulating effect of cystamine]. Radiobiologiia.

[CR134] Saksonov PP (1975). Protection against radiation (biological, pharmacological, chemical, physical). Foundation of Space Biology and Medicine, Vol. 3.

[CR135] Savoye C, Swenberg C, Hugot S, Sy D, Sabattier R, Charlier M, Spotheim-Maurizot M (1997). Thiol WR-1065 and disulphide WR-33278, two metabolites of the drug ethyol (WR-2721), protect DNA against fast neutron-induced strand breakage. Int J Radiat Biol.

[CR136] Selye H (1985). The nature of stress. Basal Facts.

[CR137] Shannon MF, Coles LS, Vadas MA, Cockerill PN (1997). Signals for activation of the GM-CSF promoter and enhancer in T cells. Crit Rev Immunol.

[CR138] Shashkov VS, Anashkin OD, Suvorov NN, Manaeva IA (1971). [Efficacy of serotonin, mexamine, AET and cystamine with repeated administration after γ-irradiation]. Radiobiologiia.

[CR139] Sieber F, Muir SA, Cohen EP, Fish BL, Mäder M, Schock AM, Althouse BJ, Moulder JE (2011). Dietary selenium for the mitigation of radiation injury: effects of selenium dose escalation and timing of supplementation. Radiat Res.

[CR140] Singh VK, Brown DS, Kao TC (2010). Alpha-tocopherol succinate protects mice from gamma-radiation by induction of granulocyte-colony stimulating factor. Int J Radiat Biol.

[CR141] Singh VK, Grace MB, Parekh VI, Whitnall MH, Landauer MR (2009). Effects of genistein administration on cytokine induction in whole-body gamma irradiated mice. Int Immunopharmacol.

[CR142] Sklobovskaia IE, Zhavoronkov LP, Dubovik BV (1984). Effect of the inhibition of prostaglandin biosynthesis on the hematopoietic status of irradiated mice. Radiobiologiia.

[CR143] Skrede S (1966). Effect of cystamine and cysteamine on the adenosine-triphosphotase activity and oxidative phosphorilation of rat liver mitochondria. Biochem J.

[CR144] Smirnova IB, Dontsova GV, Konstantinova MM, Rakhmanina ON (1984). Radiomodifying effect of serotonin on the cells of the hematopoietic system. Radiobiologiia.

[CR145] Smirnova IB, Dontsova GV, Konstantinova MM, Rakhmanina ON (1984). Characteristics of the post-radiation reaction of hematopoietic tissue with adrenaline use. Radiobiologiia.

[CR146] Smith WW, Budd RA, Cornfield J (1966). Estimation of radiation dose-reduction factor for beta-mercaptoethylamine by endogenous spleen colony counts. Radiat Res.

[CR147] Smith WW, Alderman IM, Gillespie RE (1958). Hematopoietic recovery induced by bacterial endotoxin in irradiated mice. Am J Physiol.

[CR148] Speciale A, Chirafisi J, Saija A, Cimino F (2011). Nutritional antioxidants and adaptive cell responses: an update. Curr Mol Med.

[CR149] Spotheim-Maurizot M, Franchet J, Sabattier R, Charlier M (1991). DNA radiolysis by fast neutrons. II. Oxygen, thiols and ionic strength effects. Int J Radiat Biol.

[CR150] Stone HB, Moulder JE, Coleman CN, Ang KK, Anscher MS, Barcellos-Hoff MH, Dynan WS, Fike JR, Grdina DJ, Greenberger JS, Hauer-Jensen M, Hill RP, Kolesnick RN, Macvittie TJ, Marks C, McBride WH, Metting N, Pellmar T, Purucker M, Robbins ME, Schiestl RH, Seed TM, Tomaszewski JE, Travis EL, Wallner PE, Wolpert M, Zaharevitz D (2004). Models for evaluating agents intended for the prophylaxis, mitigation and treatment of radiation injuries. Report of an NCI Workshop, December 3-4, 2003. Radiat Res.

[CR151] Strelkov RB, Briantseva LA, Ziia AV (1974). Possibility of practical use of a gaseous hypoxic mixture for prevention of radiation injuries. Radiobiologiia.

[CR152] Strelkov RB, Mardynskiĭ IS, Zakoshchikov KF, Firsova PP (1985). Vopr Onkol.

[CR153] Titov BA, Zherebchenko GP, Znamenskiĭ VV, Terekhov AV (1977). [Radioprotective properties of hypoxia in conditions of administration of antihypoxic agents]. Radiobiologiia.

[CR154] Travis EL (1984). The oxygen dependence of protection by aminothiols: implications for normal tissues and solid tumors. Int J Radiat Oncol Biol Phys.

[CR155] Travis EL, Fang MZ, Basic I (1988). Protection of mouse bone mar-row by WR-2721 after fractionated irradiation. Int J Radiat Oncol Biol Phys.

[CR156] Trog D, Bank P, Wendt TG, Koscielny S, Beleites E (1999). Daily amifostine given concomitantly to chemoradiation in head and neck cancer. A pilot study. Strahlentherapie Oncol.

[CR157] Turner ND, Braby LA, Ford J, Lupton JR (2002). Opportunities for nutritional amelioration of radiation-induced cellular damage. Nutr.

[CR158] Ullmann K, Wiencierz AM, Müller C, Thierbach R, Steege A, Toyokuni S, Steinberg P (2008). A high-throughput reporter gene assay to prove the ability of natural compounds to modulate glutathione peroxidase, superoxide dismutase and catalase gene promoters in V79 cells. Free Radic Res.

[CR159] Ushakov IB, Vasin MV (2011). Radiation protectors within the radiation safety system for extended duration exploration missions. Aviakosm Ekolog Med.

[CR160] Vacek A, Rotkovska D (1964). On the protective effect of 2,4-dinitrophenol. Int J Radiat Biol.

[CR161] van den Brenk H, Moore R (1959). Effect of high oxygen pressure on the protective action of cystamine and 5-hydroxytryptamine in irradiated rats. Nature.

[CR162] van den Brenk H, Haas M (1961). Studies on the mechanisms of chemical radiation protection in vivo. 1. 5-hydroxytryptamine in relation to effect of antimetabolites, antagonists and releasing agents. Int J Radiat Biol.

[CR163] van den Brenk H, Jamieson D (1962). Studies of mechanisms of chemical radiation protection in vivo. II. Effect of pressure oxygen on radioprotection in vivo and its relation to “Oxygen poisoning”. Int J Radiat Biol.

[CR164] van der Meer C, van Bekkum DW (1959). The mechanism of radiation protection by hystamine and other biological amines. Int J Radiat Biol.

[CR165] van der Meer C, van Bekkum D (1961). A study on the mechanism of radiation protection by 5-hydroxytryptamine and tryptamine. Int J Radiat Biol.

[CR166] Vasin MV, Antipov VV (1972). Radioprotective efficacy of cystamine at repeated administration before gamma-irradiation. Radiobiologiia.

[CR167] Vasin MV, Antipov VV, Chernov GA, Abramov MM, Gavrilyuk DN, L’vova TS, Suvorov NN (1997). The role of the vasoconstrictor effect in realizing the radioprotective properties of indralin in experiments on dogs. Radiats Biol Radioecol.

[CR168] Vasin MV, Antipov VV, Suvorov NN, Abramov MM, Gorelova NV (1984). Characteristics of the role of the hydroxyl group in serotonin in the pharmacological and antiradiation effect of serotonin. Radiobiologiia.

[CR169] Vasin MV, Chernov GA, Antipov VV (1997). Width of radiation protective effects of indralin in comparative studies using different animal species. Radiats Biol Radioecol.

[CR170] Vasin MV, Chernov GA, Koroleva LV, L'vova TS, Abramov MM, Antipov VV, Suvorov NN (1996). Mechanism of the radiation-protective effect of indralin. Radiats Biol Radioecol.

[CR171] Vasin MV, Chernov IN, Semenova LA (1991). Antiradiation proper-ties of radioprotectors, immunomodulators and agents affecting tissue metabolism in fractionated irradiation. Radiobiologiia.

[CR172] Vasin MV, Davydov BI, Antipov VV (1971). Comparative elimination of radiation-protective and toxic effects of cystamine. Radiobiologiia.

[CR173] Vasin MV, L’vova TS, Antipov VV, Dadydov BI (1977). Radio-protective efficacy of cystamine and mexamine on mice hematopoietic stem cells in vivo. Radiobiologiia.

[CR174] Vasin MV, L’vova TS, Antipov VV, Davydov BI (1979). Radiosensitivity of animals irradiated in an altered gaseous environment. 1. The effect of breathing normobaric pure oxygen during irradiation on body radioresistance and the antiradiation effectiveness of radioprotectors. Radiobiologiia.

[CR175] Vasin MV, Saksonov PP, Shashkov VS, Antipov VV (1970). Relation between the anti-radiation activity of aminothiols (cystamine), the dose of the preparation and the duration of its use under various conditions of gamma-irradiation. Radiobiologiia.

[CR176] Vasin MV, Semenov LF, Suvorov NN, Antipov VV, Ushakov IB, Ilyin LA, Lapin BA (2014a). Protective effect and the therapeutic index of indralin in juvenile rhesus monkeys. J Radiat Res.

[CR177] Vasin MV, Suvorov NN, Abramov MM, Gordeev EN (1987). Changes in the therapeutic spectrum with respect to the pharmacological and radioprotective activity after O-alkylation of serotonin and 5(2-hydroxyethoxytryptamine). Radiobiologiia.

[CR178] Vasin MV, Ushakov IB, Koroleva LV, Antipov VV (1999). The role of cell hypoxia in the effect of radiation protectors. Radiats Biol Radioecol.

[CR179] Vasin MV, Ushakov IB, Korovkina EP, Kovtun VI (2013). Effect of α_1_-adrenomimetic indralin on oxygen consumption by bone marrow cells in vitro. Bull Exp Biol Med.

[CR180] Vasin MV, Ushakov IB, Kovtun VY, Komarova SN, Semenova LA (2006). Effect of radioprotector indralin on carboplatinum hemotoxicity. Bull Exp Biol Med.

[CR181] Vasin MV, Ushakov IB, Kovtun VI, Komarova SN, Semenova LA, Galkin AA (2004). Comparative effectiveness of antioxidant melatonin and radioprotectors indralin and phenylephrine in local radiation injuries. Radiats Biol Radioecol.

[CR182] Vasin MV, Ushakov IB, Kovtun VI, Komarova SN, Semenova LA, Galkin AA, Afanas'ev RV (2008). Radioprotective properties of a radioprotector of emergency action indralin at its administration after irradiation in conditions of local shielding of a rat abdomen. Radiats Biol Radioecol.

[CR183] Vasin MV, Ushakov IB, Kovtun VI, Komarova SN, Semenova LA (2004). Effect of melatonin, ascorbic acid, and succinic acid on the cumulative toxic effect of repeated treatment with gammafos (amifostine). Bull Exp Biol Med.

[CR184] Vasin MV, Ushakov IB, Kovtun VI, Komarova SN, Semenova LA, Koroleva LV, Galkin AA, Afanas'ev RV (2008). The characteristic of radioprotective properties of a radioprotectant B-190 at its administration after radiation. Radiats Biol Radioecol.

[CR185] Vasin MV, Ushakov IB, Kovtun VI, Semenova LA, Koroleva LV, Galkin AA, Afanas'ev RV (2014b). The targets for radioprotective and mitigatory action of radio-protector indralin. J Radioprot Res.

[CR186] Vasin MV, Ushakov IB, Kovtun VY, Semenova LA, Koroleva LV, Galkin AA, Afanas'ev RV (2014). Therapeutic effect of long-term melatonin treatment on the course and fatal outcome of modeled acute radiation sickness. Bull Exp Biol Med.

[CR187] Vasin MV, Ushakov IB, Semenova LA, Kovtun VI (2001). Pharmacologic analysis of the radiation-protecting effect of indraline. Radiats Biol Radioecol.

[CR188] Vasin MV (1986). Comparative characteristics of the modification of radiosensitivity of mice and rats by a hypoxic mixture. Radiobiologiia.

[CR189] Vasin MV (1999). Classification of agents of radiation damage prophylaxis as formation of conceptual basis of modern radiation pharmacology. Radiats Biol Radioecol.

[CR190] Vasin MV (2012). The Non‐uniformity of the Absorption of Ionizing Radiation Energy in the Body Potentiates the Radioprotective Effects of Drugs: Influence on the Post‐radiation Recovery of Radiosensitive Tissues with High‐Dose Total‐Body Irradiation Med. Hypotheses Res.

[CR191] Vasin MV (2014a). Comments to the Mechanism of Protective and Pharmacological Action of Radioprotectors from the Family of Aminothiols. J Radioprot Res.

[CR192] Vasin MV (2014b). Bioflavonoids as important component of biological protection from ionizing radiation. Food Nutr Sci.

[CR193] Vaughan AT, Grdina DJ, Meechan PJ, Milner AE, Gordon DJ (1989). Conformational changes in chromatin structure induced by the radioprotective aminothiol WR 1065. Br J Cancer.

[CR194] Vladimirov VG, Libikova NI (1970). Cystamine effect to oxidative phosphorilation in liver mitochondria. Farmakol Toksikol.

[CR195] Voĭtkevich ND, Palyga GF (1974). Antiradiation effect of mexamine. Med Radiol (Mosk).

[CR196] Wardman P, Dennis MF, Stratford MR, White J (1992). Extracellular: intracellular and subcellular concentration gradients of thiols. Int J Radiat Oncol Biol Phys.

[CR197] Watanabe K, Taniguchi M, Miyoshi M, Shimizu H, Imoto T, Sato K, Watanabe T (2006). Effects of central injection of angiotensin-converting-enzyme inhibitor and angiotensin type 1 receptor antagonist on the brain NF-kappaB and AP-1 activities of rats given LPS. Peptides.

[CR198] Weiss JF, Landauer MR (2009). History and development of radiation-protective agents. Int J Radiat Biol.

[CR199] Westermann J, Reich G, Kopp J, Haus U, Dörken B, Pezzutto A (2001). Granulocyte/macrophage-colony-stimulating-factor plus interleukin-2 plus interferon alpha in the treatment of metastatic renal cell carcinoma: a pilot study. Cancer Immunol Immunother.

[CR200] Wu SG, Miyamoto T (1990). Radioprotection of the intestinal crypts of mice by recombinant human interleukin-1 alpha. Radiat Res.

[CR201] Yuhas JM, Proctor JO, Smith LH (1973). Some pharmacologic ef-fects of WR-2721: their role in toxicity and radioprotection. Radiat Res.

[CR202] Zhang J, Tucholski J, Lesort M, Jope RS, Johnson GV (1999). Novel bimodal effects of the G-protein tissue transglutaminase on adrenoreceptor signaling. Biochem J.

[CR203] Zhao CL, Xiu Y, Ashton J, Xing L, Morita Y, Jordan CT, Boyce BF (2012). Noncanonical NF-κB signaling regulates hematopoietic stem cell self-renewal and microenvironment interactions. Stem Cells.

[CR204] Zhao W, Robbins ME (2009). Inflammation and chronic oxidative stress in radiation-induced late normal tissue injury: therapeutic implications. Curr Med Chem.

[CR205] Zheng S, Newton GL, Gonick G, Fahey RC, Ward JF (1988). Radioprotection of DNA by thiols: relationship between the net charge on a thiol and its ability to protect DNA. Radiat Res.

[CR206] Zheng S, Newton GL, Ward JF, Fahey RC (1992). Aerobic radioprotection of pBR322 by thiols: effect of thiol net charge upon scavenging of hydroxyl radicals and repair of DNA radicals. Radiat Res.

[CR207] Zherebchenko PG, Suvorov NN (1963). On the relation between the radioprotective and vasoconstrictive action of indolylalkylamines. Radiobiologies.

[CR208] Zhu W, Xu J, Ge Y, Cao H, Ge X, Luo J, Xue J, Yang H, Zhang S, Cao J (2014). Epigallocatechin-3-gallate (EGCG) protects skin cells from ionizing radiation via heme oxygenase-1 (HO-1) overexpression. J Radiat Res.

